# Rethinking the relationship between ambulatory activity and falls in long-term care: risk versus reward

**DOI:** 10.1093/gerona/glaf197

**Published:** 2025-09-08

**Authors:** Ríona Mc Ardle, Lynne Taylor, Silvia Del Din, Lynn Rochester, Ngaire Kerse, Jochen Klenk

**Affiliations:** Translational and Clinical Research Institute, Newcastle University, Newcastle Upon Tyne, United Kingdom; School of Population Health, Faculty of Medical and Health Sciences, University of Auckland, Auckland, New Zealand; Translational and Clinical Research Institute, Newcastle University, Newcastle Upon Tyne, United Kingdom; Translational and Clinical Research Institute, Newcastle University, Newcastle Upon Tyne, United Kingdom; School of Population Health, Faculty of Medical and Health Sciences, University of Auckland, Auckland, New Zealand; Institute of Epidemiology and Medical Biometry, Ulm University, Ulm, Germany; Department of Clinical Gerontology, Robert-Bosch-Hospital, Stuttgart, Germany

**Keywords:** Residential facilities, Long-term care, Falls, Walking, Accelerometry

## Abstract

**Background:**

Ambulatory older residents in long-term care (LTC) have the highest risk of falling. However, the relationship between ambulatory activity (steps per day) and fall risk in LTC is unclear. This study examined whether baseline daily step count, functional capacity and cognitive function predicted falls in LTC residents, and whether functional capacity modified the relationship between step count and fall risk.

**Methods:**

Two hundred seventy-six LTC residents from New Zealand-based Staying UpRight randomized controlled trial were included (age: 84 ± 7 years; 61% female). Baseline daily step count was derived from a lumbar-based accelerometer (3589 ± 2379 steps). Fall rates were calculated from facilities’ falls reports (6 ± 18 falls). Residents were categorized as Moderate (*n* = 71) or Low functional capacity (*n *= 205) based on Short Physical Performance Battery scores. The Montreal Cognitive Assessment assessed cognition (15 ± 6). Quasi-Poisson generalized linear models explored associations between steps, cognition, and functional capacity with fall rates, including interactions between capacity and steps. The relative risk of falling and fall-related injuries was estimated between activity levels.

**Results:**

Key results showed a significant interaction (*P* = .036), indicating that only the Moderate functional capacity group had a positive association between steps and fall rates. The Moderate group had a ∼23%-24% and ∼6% higher relative risk of falls and fall-related injuries, respectively, with higher activity, while the Low group showed a lower risk of falls (∼2.7%-3.9%) and falls-related injuries (2%-4%). Cognitive function was not associated with falls.

**Conclusions:**

Findings suggest that higher exposure to ambulatory activity is related to greater falls risk but not falls-related injuries only among residents with moderate functional capacity. This stratification should be considered when shaping falls prevention policies.

## Introduction

With an aging population, the demand for long-term care (LTC) is rising.^1^ LTC is defined as full-service support with functional activities and necessities in facilities such as rest, care, and nursing homes.^1–3^ LTC residents have a high risk of noncommunicable diseases and mobility issues, accelerating disability and dependency.^4^^,^^5^ Promoting mobility-related activities, such as ambulatory activity, can support maintenance of physical condition and decelerate further disability and dependency. These activities also increase quality of life, social togetherness, and autonomy in LTC residents.^6^ Despite the benefits, routine ambulatory activity is believed to increase exposure to situations where falls occur, highlighting a critical safety issue.^7–10^ Falls are a significant concern within LTC, occurring 2-4x more frequently than in older adults of similar ages within the community, and leading to injuries, hospitalization and death.^11–14^ The recent World Guidelines for Falls Prevention and Management for Older Adults recommends that all LTC residents are considered at high risk of falls.^15^ Therefore, fall prevention is a key priority and acknowledged indicator of quality care, creating a dilemma between encouraging mobility while maintaining safety.^7–10^

Ambulatory activity can be objectively and precisely measured in LTC using wearable technologies.^16–18^ These objective measures demonstrate that LTC residents engage in low volumes of ambulatory activity, particularly in those receiving a higher level of care (ie, 24-hour nursing care) and with lower functional capacity.^16^^,^^17^^,^^19^ Beyond these objective studies, ambulatory activities can be restricted due to environmental, social, and policy constraints, with a particular emphasis on a “safety first” strategy.^20–23^ For example, care staff can be seen as gatekeepers to mobility-related activities and hold major concerns regarding the relationship between ambulatory activities and increased fall risk.^21^ This leads to a collective protective culture within organizations, with safety, in the form of not encouraging ambulatory activity, considered a higher priority than residents’ autonomy.^21^^,^^22^ However, despite care staff’s beliefs, the relationship between ambulatory activity and falls is unclear; video capture of falls in LTC has indicated that the highest occurrence of falls occurs during transfers and the lowest during ambulatory activities.^24^^,^^25^

To address this risk vs reward conundrum presented by care staff, we need to understand the relationship between ambulatory activity and fall rates in LTC. As low functional capacity and impaired cognition are considered key risk factors for falls in LTC, it is important to account for their influence on this relationship.^26^^,^^27^ This will support empirical decision-making regarding the safety risk of promoting ambulatory activities for supporting residents’ quality of life and autonomy. Additionally, improving our understanding of falls-free walking—the time one spends walking without experiencing a fall—could help identify safe levels of walking in LTC residents. Therefore, we propose 2 key aims: (1) To examine the association between baseline daily step count, functional capacity, and cognitive function with prospective fall rates in long-term care residents, with a particular focus on ambulatory activity; (2) to explore whether functional capacity modifies the relationship between daily step count and fall rates by considering the interaction between baseline step count and functional capacity. We also conducted a descriptive analysis to estimate the fall-free walking exposure (steps and days without falls) and relative fall risk across discrete activity levels to provide contextual insight into potential thresholds of safe ambulatory activity in this population.

## Methods

### Participants

Ambulant residents (ie, able to walk and transfer with or without a walking aid, independently or with supervisory assistance), aged 65 years or older were recruited from 24 LTC facilities in New Zealand as part of the Staying UpRight RCT^28^ (Australian New Zealand Clinical Trials Registry ACTRN12618001827224). Participants were receiving hospital-level (24-hour care by, or under supervision of a nurse) or rest-home level (24-hour health-related care but not nursing care).^29^ Residents receiving dementia-level care (ie, rest-home level care in a secure environment to minimize dementia-related risks), psychogeriatric care, respite or palliative care, or those who were acutely unwell or immobile (ie, unable to mobilize without assistance, permanently bed-bound, or reliant on a wheelchair) were excluded from the study. Ethics was provided by the New Zealand Health and Disability Ethics Committee (NZHDEC 18/NTB/151), and consent procedures have previously been described.^28^^,^^30^

### Demographic and clinical outcomes

Demographic information for participants included age and sex. The Short Physical Performance Battery (SPPB) measured functional capacity,^31^ while the Montreal Cognitive Assessment (MoCA) assessed cognitive function.^32^

### Fall and descriptors

Fall rates for the 6 months prior to study commencement and for the study period (ie, the intervention period) were calculated using audits of facilities’ falls incident reports which included information on the date, time, location, whether the fall was witnessed and whether any injuries were sustained. Participants were characterized as “fallers prior to the study” if they had had at least one fall in the 6 months before study commencement, and a “study faller” if they had a fall during their time within the study. Injurious falls were defined as any fall which led to a fall-related soft tissue and bony injuries, including those requiring and not requiring hospitalization, as reported by the falls incident reports.

A maximum value of 10 was assigned to the number of falls to limit the effect of extreme variance in statistical models.^33^ For clarity, 33 participants fell more than 10 times during the study.

### Assessment of ambulatory activity

LTC residents wore a small body-worn accelerometer continuously for up to 7 days (Axivity AX3; 23 × 32.5 × 7.6 mm, 11 g; 100 Hz, range ± 8 g, memory: 512 M) on the fifth lumbar vertebra on the lower back at baseline before the study intervention. The accelerometer was affixed onto the skin using a double-sided hydrogel adhesive and a hypoallergenic plaster (Hypafix BSN Medical Limited). Data processing methods have previously been described.^17^ Daily step count, defined as the step count over 24 h, was derived as the primary exposure outcome.

### Considerations for inclusion of data

To reduce noise in the data, we applied a minimum bout duration of 10 s and any period of rest which was ≥2.5 s was considered resting time.^34^ Additionally, we included participants if they had ≥2 days of continuous ambulatory activity data collected, as this is the minimum number of days required to reliably quantify our primary outcome (ie, step count) across different care environments, based on Buckley et al.^35^

### Data analysis

To address Aim 1, a generalized linear model with a quasi-­Poisson family and offset for observation time was employed to assess associations between study fall rate and baseline daily step count, accounting for functional capacity (SPPB) and cognitive function (MoCA) while controlling for care level (rest home, hospital). Age, sex, and prior falls status were included as covariates in a sensitivity analysis (see Table S1) but were excluded from the primary model to preserve parsimony and avoid over-adjustment. Although this randomized control trial encompassed an exercise intervention to reduce falls risk in LTC, the intervention had no effect on the main outcomes employed in this analysis; a sensitivity analysis was performed to test for the possible effect of intervention group assignment on results (Table S2).^30^

To address Aim 2, we investigated whether baseline functional capacity modified the relationship between daily step count and prospective fall rates. We stratified participants by their capacity levels: Moderate (SPPB: 7-12) and Low (SPPB: 1-6), aligned with SPPB clinical cutoff scores.^17^^,^^36^ Significant differences for volume of ambulatory activity have previously been reported between these groups.^17^ We tested for effect modification using a generalized linear model with a quasi-Poisson family, offset for observation time. The model included main effects for daily step count (continuous) and functional capacity group (Low vs Moderate), as well as a step count × functional capacity interaction term. This allowed us to evaluate whether the association between ambulatory activity and fall rates differed by functional capacity level. Additional covariates of age, gender, and prior faller status were included in a sensitivity analysis (Table S3). To support interpretation of the interaction model, we also present baseline characteristics stratified by functional capacity group (Table 1), using Mann–Whitney *U* tests for continuous variables and Pearson’s chi-square tests for categorical variables.

**Table 1.** Participant characteristics, presented for the overall group (relevant to Aim 1) and as stratified by baseline functional capacity (relevant to Aim 2).

	**Overall**, *N* = 276	**Moderate functional capacity**, *N* = 71	**Low functional capacity**, *N* = 205	***P* value** ^2^
**Care level**				.12
** Hospital**	115 (42%)	24 (34%)	91 (44%)	
** Rest home**	161 (58%)	47 (66%)	114 (56%)	
**Age**	84 (7)	83 (8)	85 (7)	**.032**
**Gender**				0.7
** Female**	169 (61%)	42 (59%)	127 (62%)	
** Male**	107 (39%)	29 (41%)	78 (38%)	
**MoCA (0-30)**	15 (6)	17 (6)	15 (6)	**.042**
** Not complete (*n*)**	13	2	11	
**SPPB (0-12)**	4.82 (2.60)	8.38 (1.46)	3.59 (1.56)	**<.001**
**Faller prior to study**				**<.001**
** Faller**	103 (37%)	13 (18%)	90 (44%)	
** Non-faller**	173 (63%)	58 (82%)	115 (56%)	
**Study faller**				.070
** Study faller**	198 (72%)	45 (63%)	153 (75%)	
** Study non-faller**	78 (28%)	26 (37%)	52 (25%)	
**Falls prior to study (*n*)**	1.03 (2.59)	0.28 (0.70)	1.29 (2.94)	**<.001**
**Study falls (*n*)**	6.1 (17.7)	3.3 (9.5)	7.0 (19.7)	**<.001**
**Study injurious falls (*n*)**	1.08 (2.16)	0.49 (1.64)	1.29 (2.29)	**<.001**
**Treatment**				.5
** Flex n stretch**	129 (47%)	36 (51%)	93 (46%)	
** Staying upright**	143 (53%)	35 (49%)	108 (54%)	
**Observation time (days)**	582 (263)	572 (253)	586 (267)	.7
**Steps per day (*n*)**	3589 (2379)	4586 (3075)	3243 (1980)	**.002**

The variables “Falls prior to the study (*n*),” “study falls (*n*),” “study injurious falls (*n*)” refer to the mean number of falls across participants. Bold p values highlight statistically significant between-group differences.

Abbreviations: MoCA, Montreal Cognitive Assessment; SPPB, Short Physical Performance Battery; treatment, intervention type allocated in Staying Upright.

**Table 2.** Predictors of fall rate during study duration based on quasi-Poisson regression models, considering daily step count, functional capacity, and cognitive function while controlling for care level (*n* = 276).

Term	Log estimate (β)	Std. error	*z* value	*P* value	Rate ratio (exp(β))	95% CI lower	95% CI upper
**(Intercept)**	−4.525	0.229	−19.751	**<.001**	0.011	0.007	0.017
**Steps per day (per 1000 steps)**	0.031	0.031	0.970	.333	1.031	0.968	1.095
**SPPB**	−0.147	0.030	−4.875	**<.001**	0.863	0.812	0.915
**MoCA**	0.002	0.011	0.137	.891	1.002	0.980	1.023
**Care level: rest home vs hospital (ref)**	−0.131	0.139	−0.937	.350	0.878	0.669	1.156

Rate ratios >1 indicate increased fall risk; rate ratios <1 indicate reduced fall risk. Bold p values denotes statistical significance.

Abbreviations: MoCA, Montreal Cognitive Assessment; SPPB, Short Physical Performance Battery.

**Table 3.** The interaction between functional capacity level and steps per day when predicting fall rates during the study (*n* = 276).

Term	Log estimate (β)	Std. error	*z* value	*P* value	Rate ratio (exp(β))	95% CI lower	95% CI upper
**Intercept (Moderate capacity, 0 steps)**	−6.115	0.342	−17.903	**<.001**	0.002	0.001	0.004
**Functional capacity: Low vs moderate (ref)**	1.206	0.373	3.235	**.001**	3.339	1.647	7.130
**Steps per day (per 1000 steps)**	0.107	0.053	2.007	**.046**	1.113	0.998	1.231
**Functional capacity × Steps per day**	−0.140	0.066	−2.109	**.036**	0.870	0.765	0.992

Rate ratios >1 indicate increased fall risk; rate ratios <1 indicate reduced fall risk. Bolded p values denotes statistical significance.

**Figure 1.**
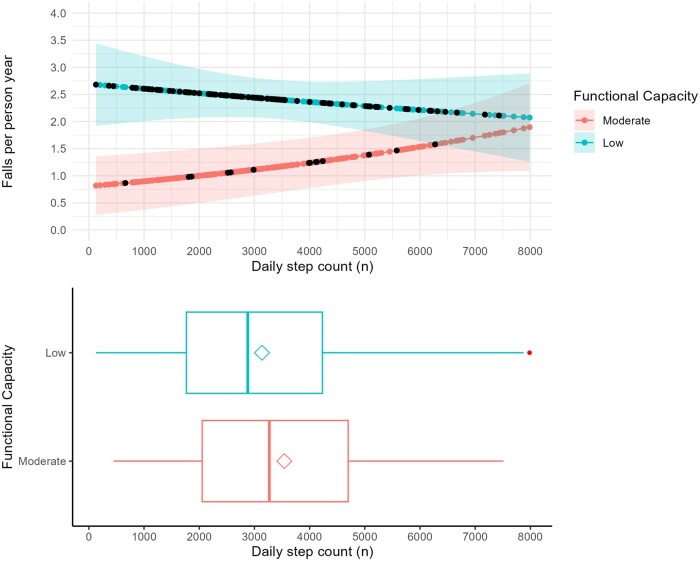
Modeled interaction between functional capacity and daily step count on predicted falls per person-year in long-term care residents (*n* = 276). Predicted fall rates were estimated from a quasi-Poisson regression model including the interaction between physical capacity and daily step count. Ribbons represent 95% confidence intervals. Black dots indicate model-predicted fall rates for individuals who experienced at least one injurious fall. Injurious falls were not modeled separately due to limited event counts, but are shown descriptively for comparison.

Injurious falls were not modeled separately due to the small number of events. Instead, predicted falls per person-year were calculated for individuals who experienced at least one injurious fall, using the main model of overall falls, and plotted on the same scale for visual comparison (Figure 1).

To aid clinical interpretation, we calculated a derived outcome representing the average number of steps a participant could take before experiencing a fall–referred to as “falls-free steps.” These values were calculated by applying predicted falls per year from the main model (Aim 1) to fixed, illustrative levels of daily step count (eg, 2000, 4000, 6000 steps/day) using the formula: Estimated falls-free steps were calculated by activity level (ie, 2000, 4000, 6000 steps per day) as follows:


$$\frac{\mathrm{Daily}\;\mathrm{steps}*365\;\mathrm{days}}{\mathrm{Predicted}\;\mathrm{falls}\;\mathrm{per}\;\mathrm{year}\;}.$$

Estimated falls-free days were calculated by normalizing fall-free walking to the activity levels. These estimates were intended to contextualize relative risk across activity levels. Identical methods were used to estimate injurious falls-free steps and days.

## Results

### Demographic information

Two hundred seventy-six participants were included in the analysis; 58% were receiving rest home level care, and 42% were hospital level care. The mean age was 84 ± 7 years, and 61% were female. On average, residents showed moderate cognitive decline (MoCA: 15 ± 6) and low functional capacity (SPPB: 5 ± 3). The average follow-up period for the main study was 20.8 ± 9.3 months; for the subset in this analysis, it was 19.1 ± 8.2 months. Over the study period, 61 participants died, 13 were discharged from the facility or entered palliative care, and 15 requested to be withdrawn from the study. The average number of days participants wore the accelerometer was 6 ± 1. Participants took an average of 3589 steps per day, ranging from 128 to 13807 steps. Table 1 provides all demographic information. During the study, 72% of participants were reported to have fallen.

### Aim 1: Examining the association between baseline step count, functional capacity, and cognitive function and prospective fall rates

Neither daily step count nor cognitive function significantly predicted fall rates during the study (see Table 2). Only baseline functional capacity was statistically associated with fall rates, suggesting that higher capacity is associated with fewer falls. Table S2 provides model details, which include the intervention treatment group; there was no effect for intervention.

### Aim 2: Exploring whether baseline functional capacity modifies the relationship between baseline ambulatory activity and prospective fall rates

Demographic information for the cohort stratified by baseline functional capacity scores can be found in Table 1. Participants in the Low group are older (*P* = .032), with greater cognitive impairment (MoCA 15 vs 17; *P* = .042), had a greater number of study falls (*P* < .001), and study falls leading to injury (*P* < .001) and took fewer daily steps (*P* = .002) compared to the Moderate group.

When considering the interaction between baseline functional capacity level and steps per day in predicting rate of study falls, the model indicated that participants in the Low group had a significantly higher fall rates than the Moderate (Rate ratio: 3.34, *P* < .001). Higher step counts were associated with increased fall rates (Rate ratio 1.11 per 1000 steps, *P* = .046; see Table 3). There was a statistically significant interaction between functional capacity level and steps per day, indicating that the effect of daily steps on falls varies by physical capacity level (*P* = .036; see Figure 1); a greater step count was associated with increased fall rates only in the Moderate group, but not in the Low capacity group. A sensitivity analysis revealed no effect for the intervention group (see Table S4).

**Table 4.** Predicted number of (a) falls-free steps per year and days and (b) injurious falls-free steps per year and days across activity levels within the functional capacity group (*n* = 276).

Physical capacity level	Activity level	Predicted falls per year	Falls-free steps	Falls-free days	Relative risk of falling	Predicted injurious falls per year	Injurious falls-free steps	Relative risk of injurious falls
**Moderate**	2000 steps per day	1	730 681	365		0.28	2 578 837	
**Low**	2000 steps per day	2.52	289 421	145		0.78	934 195	
**Moderate**	4000 steps per day	1.24	1 179 666	295	24%^a^	0.29	5 038 013	3.60%^a^
**Low**	4000 steps per day	2.36	618 030	155	−6% ^a^	0.75	1 934 320	−3.90%^a^
**Moderate**	6000 steps per day	1.53	1 428 404	238	23% ^b^	0.3	7 381 693	3.50%^b^
**Low**	6000 steps per day	2.21	989 805	165	−6% ^b^	0.73	3 003 867	−2.70%^b^

Falls-free steps and days were calculated as descriptive metrics based on model-predicted fall rates. Values represent average steps or days expected before a fall, conditional on modeled fall risk. Confidence intervals are not shown, as these metrics are derived rather than directly estimated. a = compared to 2000 steps, b = compared to 4000 steps.

### Contextual insights: Describing falls-free ambulatory exposure and relative falls risk across activity levels

Table 4 provides estimates for falls-free steps and days, and relative risk of falls and falls-related injuries based on discrete activity levels (ie, 2000, 4000, 6000 daily steps) across functional capacity groups. For residents with Moderate functional capacity, the relative risk of falling was ∼23%-24% higher for every additional 2000 daily steps, while the relative risk of injurious falls was greater by <4%. For residents with Low functional capacity, the relative risk of falling was 6% lower for every additional 2000 daily steps, while the relative risk of injurious falls was <4% lower.

### Contextual insights: Describing circumstances in which falls occurred during the study

Fall location was recorded for 1582 falls involving 194 participants. Most falls occurred in residents’ bedrooms (64%) or adjacent bathrooms (11%), followed by dining/lounge areas (14%) and corridors, foyers, or nurses’ stations (7%). Fewer than 2% occurred outdoors (see Table S5).

## Discussion

To our knowledge, this is the first study to provide objective data on ambulatory activity and falls in LTC, producing novel evidence on the interaction between baseline daily step count and functional capacity levels in predicting falls in LTC residents. Aligned with our first aim, we demonstrated that there was no evidence of association between fall rates and baseline step count or cognitive function, with only baseline functional capacity predicting study fall rates. Addressing our second aim, we found that residents with low functional capacity had a higher baseline fall rates with a greater proportion of participants experiencing injurious falls, but a higher step count was not associated with an increase in fall rate in this group. In contrast, those with moderate functional capacity showed a lower baseline fall rates and fewer injurious fallers but demonstrated an increased fall rate with a higher baseline step count. However, the prevalence of injurious fallers in the moderate group remained low, even at high step counts. This interaction is illustrated in Table 4, which presents estimates derived from the primary interaction model, applied to fixed daily step count levels. For example, at 2000 steps per day, the estimated fall rates was 1 fall per year for the moderate group and 2.5 falls for the low group. At 6000 steps per day, the estimated rate increased to 1.5 falls per year for the moderate group, while the low group decreased slightly to 2.2. Injurious fall rates remained consistent across different step count levels for both groups.

These findings have important implications for falls prevention in LTC. Although care staff may be concerned that increasing ambulatory activity will lead to more falls, our results suggest a more nuanced picture, for which we will present some potential explanations and suggestions for clinical consideration.^5^^,^^7^^,^^10^^,^^23^^,^^25^ Residents with low functional capacity appear to fall frequently regardless of activity level, likely due to high baseline risk and limited mobility, while those with moderate functional capacity show a modest increase in fall rates when participating in greater ambulatory activity.^17^^,^^37^^,^^38^ A potential explanation is that ambulatory activity may be a proxy for broader mobility engagement, requiring more frequent participation in other high-risk movements such as transferring.^11^^,^^24^ Previous studies using video analysis have shown that most falls in LTC occur during standing or transferring activities, rather than walking.^11^^,^^24^ For those with low capacity, transferring behaviors may be inherently risky and lead to frequent falls regardless of walking volume. For those with moderate capacity, engaging in more ambulatory activity may increase exposure to those behaviors, resulting in higher fall rates as activity levels increase. However, further observation of falls circumstances would be needed to verify this proposal; although our contextual insights may support this with most reported falls occurring in bedrooms or bathrooms and few occurring outside the facilities, these activities were often not witnessed. Therefore, while we could hypothesize that falls in restricted spaces are likely to occur during non-walking or limited walking activities such as transferring, this cannot be verified in this study.

Regarding potential clinical considerations, these results highlight the importance of stratifying residents by functional capacity when designing falls prevention policies or strategies. This could be operationalized via the inclusion of a simple assessment of functional capacity in the multifactorial falls risk assessment recommended in the World Falls Prevention guidelines conducted at LTC admission.^15^ Rather than applying blanket “safety first” restrictions using a one-size-fits-all approach, stratification could help care providers make proportionate, person-centered decisions that balance fall risk with residents’ wellbeing and autonomy.^6^^,^^9^^,^^20–22^^,^^25^^,^^39^ This aligns with Iaboni et al.’s^40^ proposed palliative approach to falls, which advocates for managing risk in a way that accepts some degree of falling as tolerable, particularly if it supports meaningful activity and preserves dignity. Our findings provide a simple and practical framework (stratification by functional capacity) to support this approach. Although we are not recommending that LTC residents increase their step count, our data suggest that higher walking volumes are not associated with increased falls in residents with low functional capacity, and that injurious falls remain low in the moderate capacity group, even at higher activity levels. Exact intervention types for each group needs to be further investigated, with input from all stakeholders, including people living in LTC, their families, and LTC staff and commissioners, to ensure the balance of safety and autonomy is inclusive to all perspectives.^41^^,^^42^ These novel insights from the current study may initially help reframe how mobility is supported in LTC, not only as a potential risk, but recognizing its broader contribution to psychosocial wellbeing, autonomy, and dignity.^39^^,^^40^

Secondary findings suggest no evidence of association between cognitive function and fall rates, despite evidence in the literature of dementia as a risk factor for falls in LTC.^26^^,^^43^ Additionally, previous published findings have additionally reported no association between volume of ambulatory activity and cognitive function in this study population.^17^ It should be noted that we did not include dementia unit participants (ie, those in secure care with additional support for dementia related behaviors) in this analysis, due to the small sample size (*n* = 25) and significant heterogeneity within this group.^28^^,^^30^ However, previous work has indicated that dementia unit residents have the highest falls risk, due to the combination of wandering behaviors, continence status, mobility level, use of psychotropic medications, and behavioral symptoms.^43^ Our prior work has shown that dementia unit residents had substantially lower cognitive scores, but significantly higher ambulatory volumes compared to hospital and rest home care levels.^17^ Coupled with behavioral symptoms, this would create quite a different falls risk profile. Therefore, our findings are limited to LTC settings outside of dementia units but may assuage care staff’s anxieties regarding fall risk in cognitively impaired residents in these settings and allow them to make empirically-informed decisions regarding residents’ relative safety in relation to ambulatory activities.^27^^,^^44^

### Strengths and limitations

Strengths of this study include the use of validated, standardized accelerometry measures to capture ambulatory activity, allowing for an objective and precise dataset and comparative outcomes to multiple other cohorts; the use of clinical cutoff scores to characterize functional capacity, providing clinical applicability; and a sample drawn from multiple LTC facilities representing different care levels, functional capacity and cognitive function, which is rarely included in research in this area.^16^^,^^45^ This study has several limitations. Falls incidence reports in LTC record the time and location of falls, but falls are rarely witnessed. Context inference (eg, during transfer or ambulation) may be imprecise, and >50% of all falls lacked contextual information. Therefore, we could only provide information on the location where falls occurred rather than information regarding the activity in which it occurred (eg, walking, transferring) which limited our interpretation. Twenty-four facilities were included in this study and may bring underlying environmental and organizational differences which influence ambulatory activity; we were not able to capture these.^44^ This study reflects a New Zealand LTC context; further research should replicate this work in other countries to understand broader applicability. We aimed to recruit a broad sample of mobile aged care residents; however, those who consented to participate in the exercise trial and wear sensors may have been higher functioning. Physical capacity scores (SPPB: 4.8 ± 2.6) in our study were slightly higher than those reported in a recent observational study of aged care residents (4.1 ± 3.3).^46^ Additionally, we only included participants who could ambulate, excluding those who were most functionally impaired (eg, wheelchair bound). Although this was necessary to assess ambulatory activity, it may have led to overestimation of overall fall rates in the sample, as non-ambulatory residents are generally at lower risk of falls due to limited mobility. This dataset was derived from a randomized controlled trial; however, we have reported sensitivity analysis results considering the impact of the intervention, with no change to our interpretation of findings.^30^

## Conclusion

The effect of ambulatory activity on falls in LTC differs by functional capacity level but does not appear to have a significant effect on fall-related injuries. Key findings challenge the need to restrict routine ambulatory activity as a falls prevention strategy and highlight stratification by functional capacity as a potential avenue to safely enhance routine ambulatory activity in LTC.

## Supplementary Material

glaf197_Supplementary_Data

## Data Availability

The data supporting the findings of this study are available on request from the last author, Professor Ngaire Kerse. The data are not publicly available due to privacy or ethical restrictions.
