# Supplementary material for: Rethinking the relationship between ambulatory activity and falls in long-term care: risk versus reward
Source: J Gerontol A Biol Sci Med Sci. 2025 Sep 8;80(11):glaf197. doi: 10.1093/gerona/glaf197 (PMC12527237; doi:10.1093/gerona/glaf197)
Supplement: glaf197_Supplementary_Data [file glaf197_supplementary_data.zip › Supplementary material.docx]

# Supplementary Material

Table of contents:

1. **Table 1: Predictors of fall rate during study duration based on quasi-poisson regression models, considering daily step count, functional capacity and cognitive function while controlling for care level, age, gender and prior faller status**
2. **Table 2**: **Predictors of falls during study duration, considering ambulatory outcomes, physical capacity, cognitive impairment, care environment and intervention group**
3. **Table 3: The interaction between functional capacity level and steps per day when predicting falls rate during the study, controlling for care level, age, gender and prior faller status.**
4. **Table 4:** **The interaction between physical capacity level and steps per day when predicting falls rate during the study, considering effect of intervention group**
5. **Table 5: Description of falls by location**

**Table 1: Predictors of fall rate during study duration based on quasi-poisson regression models, considering daily step count, functional capacity and cognitive function while controlling for care level, age, gender and prior faller status**

| Term | Log Estimate (β) | Std. Error | p-value | Rate Ratio (exp(β)) | 95% CI Lower | 95% CI Upper |
| --- | --- | --- | --- | --- | --- | --- |
| (Intercept) | -5.263 | 0.876 | **<0.001** | 0.005 | 0.001 | 0.028 |
| Steps per day (per 1000 steps) | 0.017 | 0.031 | 0.572 | 1.017 | 0.957 | 1.079 |
| SPPB | -0.099 | 0.031 | **0.001** | 0.906 | 0.852 | 0.961 |
| MoCA | 0.005 | 0.011 | 0.657 | 1.005 | 0.984 | 1.026 |
| Care Level: Rest Home | -0.094 | 0.131 | 0.472 | 0.910 | 0.705 | 1.179 |
| Age | 0.010 | 0.009 | 0.311 | 1.010 | 0.991 | 1.029 |
| Gender (male) | 0.067 | 0.138 | 0.625 | 1.070 | 0.815 | 1.397 |
| Faller: Non-Faller | -0.664 | 0.140 | **<0.001** | 0.515 | 0.390 | 0.676 |

*SPPB = Short Physical Performance Battery, MoCA = Montreal Cognitive Assessment. N.B. Rate ratios >1 indicate increased fall risk; rate ratios <1 indicate reduced fall risk.*

**Table 2**: **Predictors of falls during study duration, considering ambulatory outcomes, physical capacity, cognitive impairment, care environment and intervention group**

| term | estimate | std.error | statistic | p.value |
| --- | --- | --- | --- | --- |
| (Intercept) | -4.478 | 0.252 | -17.769 | **0.000** |
| Steps per day | 0.000 | 0.000 | 0.894 | 0.372 |
| SPPB | -0.149 | 0.030 | -4.882 | **0.000** |
| MoCA | 0.001 | 0.011 | 0.104 | 0.917 |
| Care Environment: Rest Home | -0.154 | 0.141 | -1.088 | 0.277 |
| Intervention: Active group | -0.030 | 0.141 | -0.216 | 0.829 |

*SPPB = Short Physical Performance Battery, MoCA = Montreal Cognitive Assessment*

**Table 3: The interaction between functional capacity level and steps per day when predicting falls rate during the study, controlling for care level, age, gender and prior faller status.**

| Term | Log Estimate (β) | Std. Error | p-value | Rate Ratio (exp(β)) | 95% CI Lower | 95% CI Upper |
| --- | --- | --- | --- | --- | --- | --- |
| Intercept (Moderate capacity, 0 steps) | -6.349 | 0.868 | **<0.001** | 0.002 | 0.000 | 0.009 |
| Functional capacity: Low vs moderate (ref) | 0.814 | 0.342 | **0.018** | 2.256 | 1.176 | 4.505 |
| Steps per day (per 1000 steps) | 0.089 | 0.048 | 0.064 | 1.093 | 0.991 | 1.196 |
| Care Level: Rest home vs hospital (ref) | -0.042 | 0.130 | 0.747 | 0.959 | 0.744 | 1.238 |
| Age | 0.011 | 0.009 | 0.235 | 1.011 | 0.993 | 1.030 |
| Gender (malevs female (ref)) | 0.056 | 0.135 | 0.678 | 1.057 | 0.810 | 1.373 |
| Faller: Non-Faller vs faller (ref) | -0.787 | 0.137 | **<0.001** | 0.455 | 0.347 | 0.594 |
| Low capacity * Steps per day | -0.118 | 0.060 | **0.049** | 0.889 | 0.791 | 1.001 |

**Table 4:** **The interaction between physical capacity level and steps per day when predicting falls rate during the study, considering effect of intervention group**

| term | estimate | std.error | statistic | p.value |
| --- | --- | --- | --- | --- |
| (Intercept) | -6.104 | 0.356 | -17.162 | **0.000** |
| Physical capacity: Low-Very Low | 1.213 | 0.376 | 3.228 | **0.001** |
| Steps per day (n) | 0.000 | 0.000 | 1.977 | **0.049** |
| Intervention: Active Group | -0.017 | 0.145 | -0.115 | 0.909 |
| Physical Capacity Level* Steps per day | 0.000 | 0.000 | -2.119 | **0.035** |

**Table 5: Description of falls by location**

| **Location** | **Low physical capacity** | | **Moderate physical capacity** | | | **Total** | |
| --- | --- | --- | --- | --- | --- | --- | --- |
|  | **n** | **%** | **n** | **%** | **n** | | **%** |
| Resident's room | 889 | 65.5 | 127 | 56.4 | 1016 | | 64.2 |
| Bathroom/toilet | 141 | 10.4 | 34 | 15.1 | 175 | | 11.1 |
| Other resident's room | 6 | 0.4 |  |  | 6 | | 0.4 |
| Dining/lounge | 199 | 14.7 | 25 | 11.1 | 224 | | 14.2 |
| Corridors/foyer/nurses station | 88 | 6.5 | 21 | 9.3 | 109 | | 6.9 |
| Outdoors area | 23 | 1.7 | 13 | 5.8 | 36 | | 2.3 |
| Outside Facility | 11 | 0.8 | 5 | 2.2 | 16 | | 1.0 |
| **Total** | **1357** |  | **225** |  | **1582** | |  |

Notes: Low physical capacity = SPPB 0-6, Moderate physical capacity = SPPB 7-12
